# Destabilization Mechanism of Ionic Surfactant on Curcumin Nanocrystal against Electrolytes

**DOI:** 10.3390/scipharm84040685

**Published:** 2016-10-18

**Authors:** Heni Rachmawati, Annisa Rahma, Loaye Al Shaal, Rainer H. Müller, Cornelia M. Keck

**Affiliations:** 1 Pharmaceutics Research Group, School of Pharmacy, Bandung Institute of Technology, Bandung 40132, Indonesia; 2 Research Center for Nanosciences and Nanotechnology, Bandung Institute of Technology, Bandung 40132, Indonesia; annisarahma@fa.itb.ac.id; 3 Department of Pharmacy, Pharmaceutical Technology, Biopharmaceutics & NutriCosmetics, Freie Universität Berlin, Berlin 12169, Germany; losh79@gmail.com (L.A.S.); nanoteam@gmx.com (R.H.M.); cornelia.keck@pharmazie.uni-marburg.de (C.M.K.)

**Keywords:** curcumin, nanosuspension, electrolyte, particle aggregation, stabilizer, ionic surfactant

## Abstract

We have successfully developed curcumin nanosuspension intended for oral delivery. The main purpose is to improve bioavailability through enhancing its solubility. The nanoparticles were stabilized using various stabilizers, including polyvinyl pyrrolidone (PVP), polyvinyl alcohol (PVA), sodium carboxymethylcellulose (Na-CMC), d-α-tocopheryl polyethylene glycol 1000 succinate (TPGS), and sodium dodecyl sulfate (SDS). The average diameter of particles, microscopic appearance, and sedimentation of each preparation was observed and compared. Each stabilizer demonstrated a different degree of inhibition of particle aggregation under electrolyte-containing simulated gastrointestinal (GIT) fluid. Non-ionic stabilizers (PVA, PVP, and TPGS) were shown to preserve the nanosuspension stability against electrolytes. In contrast, strong ionic surfactants such as SDS were found to be very sensitive to electrolytes. The results can provide useful information for the formulators to choose the most suitable stabilizers by considering the nature of stabilizers and physiological characteristics of the target site of the drug.

## 1. Introduction

Oral delivery of active compounds is the most important target during product development due to various benefits of this route, not only for capacity of the absorption site but also for the users’ or patients’ acceptance. Despite the highest acceptability, many challenges are present when developing oral formulation of active pharmaceutical ingredient (API). Due to complexity along the gastrointestinal tract (GIT) from stomach to colon, which is characterized by different physiological pH, anatomy, and enzymatic content, the barriers for drug delivery through oral route are the most challenging compared to other dosage forms. In particular, when the API is absorbed in gastric fluid, the main digestion part of the GIT, the presence of various endogenous substances such as bile salt, electrolytes, and enzymes in that segment potentially act as barriers. The development of the pharmaceutical product hence must consider all these factors to maintain physicochemical stability and efficacy.

Curcumin, (1*E*,6*E*)-1,7-bis(4-hydroxy-3-methoxyphenyl)hepta-1,6-diene-3,5-dione, is one of the most extensively studied natural compounds. It is the major constituent of curcuma (turmeric) rhizome. In aqueous medium, curcumin exhibited three pKa values: 7.8 (pKa_1_), 8.5 (pKa_2_), and 9.0 (pKa_3_) [1]. The solubility of curcumin in water is highly limited, being practically insoluble in acidic or neutral environment but slightly soluble in alkaline medium. Curcumin is extremely susceptible to hydrolytic degradation in the presence of feruloyl methane and ferulic acid [2]. It is reported to be stable below pH 6.0. Therefore, despite the great potency of curcumin in treating various diseases, its clinical use is limited by its low solubility and stability. According to biopharmaceutical classification system (BCS), curcumin is classified as class IV compound (low permeability, low solubility) [3]. We have successfully developed a curcumin nanocrystal intended for oral delivery. The main purpose is to improve bioavailability through enhancing its solubility. Various stabilizers were applied in the formula to avoid aggregation. This report describes the ability of the stabilizers to maintain nanoscale distribution of curcumin nanocrystal when entering the gastric compartment which has low pH and contains endogenous electrolytes. This in turn will help formulators to choose the most suitable stabilizers by considering its nature and the physiological characteristics of the target site of the drug. GIT mimicking fluid was used to study the influence of surfactant on curcumin nanocrystal stability against electrolytes.

## 2. Results and Discussion

### 2.1. Effect of Stabilizer Type on Particle Size and Size Distribution

Higher surface area in nanoparticles leads to higher Gibbs free energy, causing nanoparticles to be less stable than microparticles or larger particles [4]. This must be taken into consideration in development of nanosuspension for oral routes since nanoparticles are susceptible to gastrointestinal electrolytes-induced destabilization. Therefore, a suitable stabilizer at an optimum concentration is essential in order to maintain in vivo stability of nanosuspension [4,5]. 

Sodium carboxymethylcellulose (Na-CMC), polyvinyl alcohol (PVA), and polyvinyl pyrrolidone (PVP), used in this study represented long chain macromolecular stabilizers which were adsorbed on the surface of cucumin nanocrystals and sterically stabilized the particles [4,6,7]. Effective concentration for nanocrystal stabilization is very critical [8]. Studies reported that stabilizers at low concentration often showed destabilizing effect towards nanoparticles through polymer bridging mechanism [9,10]. By using 1% of these polymeric stabilizers, the surface of the particles was suggested to be sufficiently coated by the polymer. Thermal motion in the dispersion system causes dynamic change in the polymer-coated surface of the particles. This can generate repulsive force between particles against aggregation.

### 2.2. Surface Charge of Curcumin Nanosuspension

**Figure 1:**
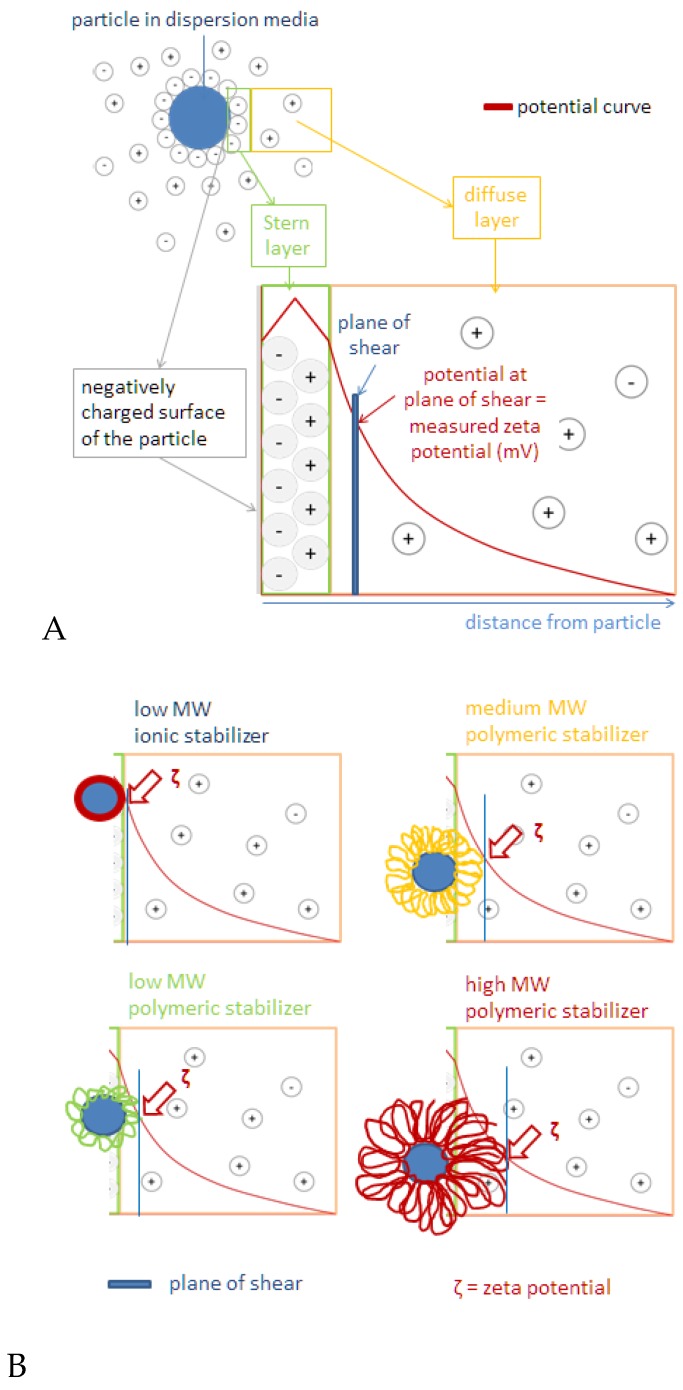
The model of zeta potential theory. (**A**) The course of the potential in the different layers; (**B**) when polymer are used for stabilization, the shear plane is shifted due to the adsorption layer resulting in decreased zeta potential.

Sodium dodecyl sulfate (SDS) at low concentration (five times lower than other stabilizers used in this study) was able to produce the highest zeta potential (ZP) value measured both in water and in dispersing medium (above −30 mV in both media). This was thought to be the result of the nature of SDS as a great electrostatic stabilizer. Quick adsorption of negatively charged alkyl chains in SDS molecules to the surface of particles results in negatively charged particle surface [11]. Our findings regarding high ZP value of nanosuspension with SDS as the stabilizer was in accordance with DLVO theory (Figure 1, left panel). Anionic head group of SDS, SO_4_^2−^, was adsorbed onto the surface of curcumin nanoparticles, resulting in stronger negative charge at the inner Helmholtz plane (IHP). As adsorption continued to occur following the Langmuir adsorption isotherm theory [12] the potential of IHP increased. This event eventually led to higher ZP in the original dispersion medium in comparison with water (−52.2 vs. −30.6 mV). 

Similar circumstances might apply to Na-stabilized nanosuspension. As described previously, Na-CMC stabilizes particles through steric and electrostatic repulsion mechanisms. The ZP value of the Na-CMC stabilized system observed in the original dispersion medium was 20 mV higher than in water. It is thought that the adsorption of carboxylic groups of Na-CMC onto the particle surface caused an increase in IHP potential and thus, the ZP increased. A higher ZP value also indicates that the Stern potential increased. This ZP value is above the limit required for physically stable dispersion systems of ~|30| mV [12]. However, particle aggregation in a Na-CMC system eventually occurred. Therefore, the ZP value is not the only parameter for nanocrystal stabilization. Furthermore, polymer bridging-aggregation by Na-CMC seemed more dominant, which explains the larger size of curcumin nanocrystal stabilized with Na-CMC. 

By contrast, steric stabilization effect provided by PVA, PVP, and d-α-tocopheryl polyethylene glycol 1000 succinate (TPGS) resulted in relatively lower ZP, being −11.3 mV in average. The ZP value of PVA-stabilized nanosuspension was the closest to zero, at −5.5 mV. When these neutral polymers are adsorbed onto the particle surface, the plane of shear is repositioned further away from the surface (Figure 1B) which leads to lower ZP value [13]. There was negligible difference of ZP value measured in aqueous medium and original dispersion medium, suggesting that there was no desorption process upon dilution with water. This stable adsorption can be explained by multi-point attachment of polymeric chains to the particle surface [14]. Unlike steric stabilizers, SDS underwent desorption process which was indicated by the decrease in ZP value by 19 mV. Multi-point attachment of Na-CMC to particle surface has been reported in several studies [15], although the affinity is relatively lower than that of other neutral polymers. Moreover, a study confirmed partial desorption of Na-CMC after dilution with water due to repulsion force between negatively charged particle surface and polymeric chains of CMC [16]. Since the stabilization of nanosuspesions by PVP, PVA, and TPGS was through a steric mechanism, low ZP in the dispersion system does not always indicate poor physical stability. 

Surface charge of dispersed particles is one of the parameters that should be considered in physical stability of the system. Measurement of zeta potential, a function of surface charge of particles, can indicate physical stability of such system. In many cases, agglomeration behavior of dispersed nano powder is highly influenced by their zeta potential [17]. Findings in zeta potential measurement in this study suggest that all five stabilizers used in this study are applicable for curcumin nanosuspension.

### 2.3. The Influence of Electrolytes on the Physical Stability of Curcumin Nanosuspension

A pre-requirement to achieve improved oral bioavailability with drug nanocrystals is physical stability without aggregation. The bioavailability decreases with increasing aggregate formation. Therefore, it is necessary to prepare nanosuspension with good physical stability. We evaluated stability of all nanosuspensions against electrolytes. 

**Figure 2:**
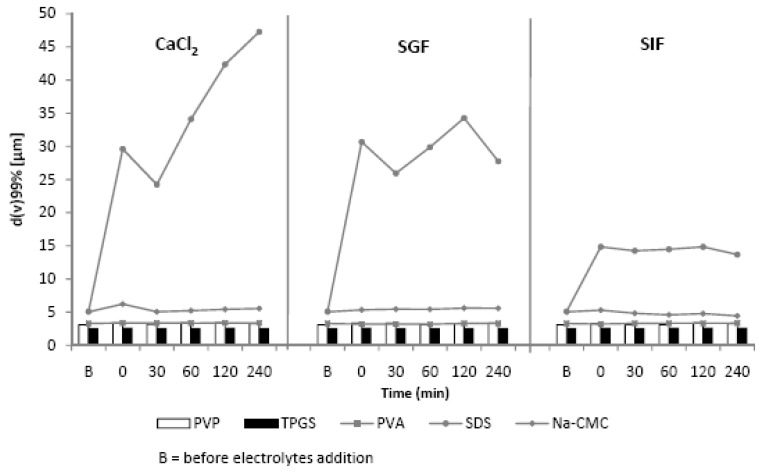
Diameter D_99_ of the five different curcumin nanosuspensions after addition of CaCl_2_ (left), simulated gastric fluid (SGF, middle) and simulated intestinal fluid (SIF, right). Measurements were performed as a function of time by laser diffractometry (LD). PVP: polyvinyl pyrrolidone; TPGS: d-α-tocopheryl polyethylene glycol 1000 succinate; PVA: polyvinyl alcohol, SDS: sodium dodecyl sulfate; Na-CMC: sodium carboxymethylcellulose.

**Figure 3:**
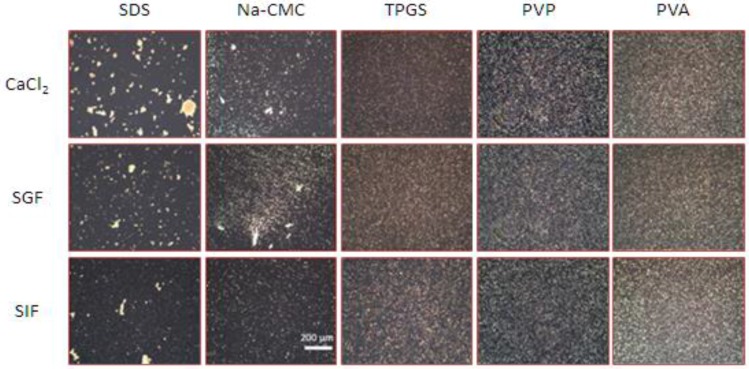
Polarized microscopy images of curcumin nanosuspension after incubation with electrolytes (160 time-magnification). Particle agglomeration was observed in SDS- and Na-CMC-stabilized nanosuspensions. Agglomerates were absent in nanosuspensions stabilized with TPGS, PVP, and PVA.

**Figure 4:**
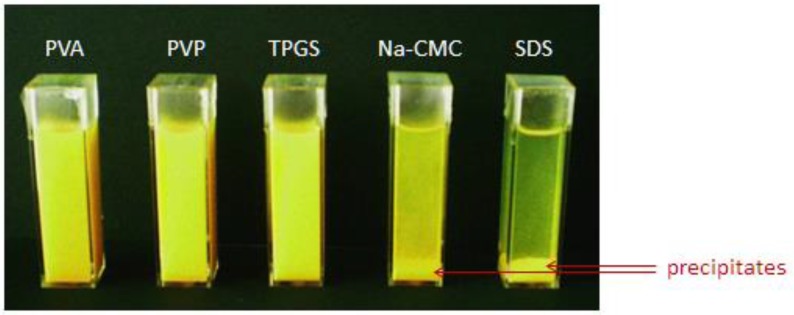
Visual observation on settling rate of curcumin nanosuspension with different stabilizers, 30 min after CaCl_2_ addition. CaCl_2_ is the most effective electrolyte in destabilizing curcumin nanosuspension preserved with SDS and Na-CMC.

The efficiency of the stabilizers to prevent particles aggregation after electrolyte challenge is clearly shown by laser diffractometry (LD) data (Figure 2), polarized light micrographs (Figure 3) and the visual settling rate examination (Figure 4). The zeta potential is directly influenced by the surface potential, electrolyte concentration and pH [18,19].

Therefore, ZP measurements were included to assess the influence of electrolytes (Table 1). As shown in Figure 2, Figure 3 and Figure 4, the presence of electrolytes clearly affected the physical stability of curcumin nanosuspension and it was stabilizer dependent. PVA, PVP, and TPGS were capable of stabilizing curcumin nanosuspension, indicated by constant D_99_ value (Figure 2).

As shown, curcumin nanosuspensions with steric stabilization are better to avoid aggregation in the presence of electrolytes. The polarized light micrographs (Figure 3) as well as the visual settling rate examination (Figure 4) confirmed the LD data (Figure 2). In contrast, nanosuspensions with electrostatic stabilization (SDS and Na-CMC) were more sensitive against electrolytes. The nanosuspension stabilized with SDS showed the most pronounced aggregation, particularly when this nanosuspension was incubated with CaCl_2_ and SGF. Thirty minutes after addition of these electrolytes, particle sedimentation was remarkably occurred. In particular, with Ca^2+^ ion, the settling down of the particle from dispersion system was clearly noted. Clearly visible sediments were observed after 30 min (Figure 4).

The effect of phosphate salt (SIF) on the stability of SDS-stabilized nanosuspension was also noticed but distinctly less compared to CaCl_2_ and SGF. This phenomenon can be correlated with the zeta potential values (Table 1). The zeta potential dropped from −50.2 mV (in original dispersion medium) to −7.3 (CaCl_2_ 150 mM) and to −19.1 mV (in SGF) but remained −45.9 mV in SIF. Two factors are suggested for this decrease: electrolyte and pH. The divalent Ca^2+^ ions are known to reduce the zeta potential much more than monovalent ions (e.g., Na^+^ or K^+^). Ca^2+^ ions tend to adsorb on particle surfaces, as reported for liposomes [18]. As a result, the surface charge will be compensated at a lower distance from the particle surface subsequently. The potential dropped faster and the diffuse layer was thinner [20]. Consequently, the ZP decreased lead to reduced nanosuspension stability. A low pH decreases the dissociation of negative charged functional groups on particle surface [21]. Thus reducing the Nernst potential and subsequently the ZP.

This extreme decrease in ZP caused a very dramatic increase in the LD data. The diameter D_99_ increased from 5 µm (before CaCl_2_ addition) to 50 µm (2 h after CaCl_2_ addition). When curcumin nanocrystal was dispersed in the medium with SGF, the effect of pH shift was the major cause for ZP reduction. The pH effect is attributed to the adsorption of H^+^ ion, which is the potential-determining ion. As the decrease ZP was moderate to −19.1 mV, smaller agglomerates were observed. Likewise, the reduction in ZP on Na-CMC stabilized nanosuspension after electrolytes addition, especially CaCl_2_ and SGF, led to nanosuspension destabilization. Despite Dν diameters did not show this phenomenon, both polarized micrographs and visual settling rate examination clearly proved this (Figure 3 and Figure 4). No significant change in Dν indicates loose agglomerates. During the LD measurement, they were de-aggregated [12]. This was in line with previous observation (Figure 2).

When a nanosuspension stabilized with Na-CMC was incubated in SIF, the ZP reduction was only moderate (to −33.1 mV), but dropped extremely to −17.6 mV in CaCl_2_ solution and to −9.8 mV in SGF (Table 1). There is a linear relationship between agglomerates size and sedimentation rate (Figure 4).

## 3. Materials and Methods

### 3.1. Materials

Curcumin from *Curcuma xanthorrhiza* rhizome was purchased from PT Phytochemindo Reksa (Bogor, Indonesia). PVA with molecular weight (MW) of 31,000 and SDS (MW = 288) were purchased from Fluka Chemica (GmbH, Darmstadt, Germany). Na-CMC (MW = 90,000) and PVP (MW = 25,000) were obtained from Sigma-Aldrich Chemie (GmbH, Darmstadt, Germany). TPGS (MW = 1500) was purchased from Eastman Chemical Company, Workington, UK. Ultra-purified water was obtained from Millipore (GmbH, Darmstadt, Germany). Other chemicals used in this study were of analytical grade.

### 3.2. Methods

#### 3.2.1. Preparation of Curcumin Nanosuspension

**Figure 5:**
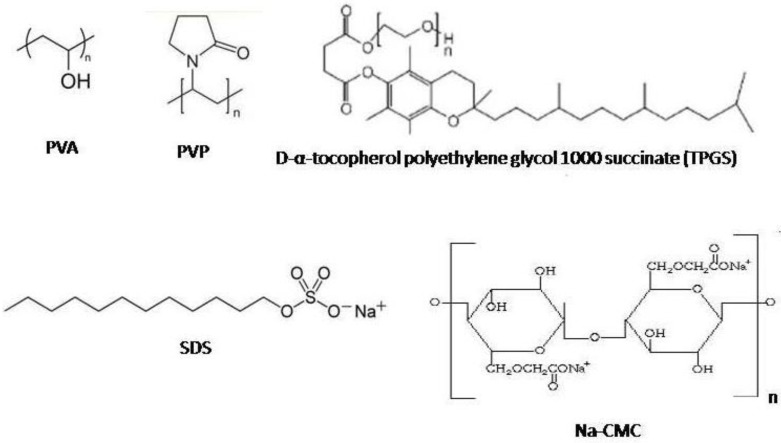
Chemical structures of stabilizers used in this study, with molecular surface charge provided.

As described in our previous report, the nanosuspension of curcumin with various stabilizers (PVA, PVP, Na-CMC, TPGS and SDS) was prepared by using high pressure homogenization (HPH) Micron Lab 40 (APV Deutchland GmbH, Germany) [22]. Both neutral and ionic stabilizers were used to prevent aggregation (Figure 5).

The particle size of curcumin nanosuspension was determined using laser diffractometry/LD (Coulter LS 230, Beckman-Coulter, Krefeld, Germany).

#### 3.2.2. Microscopic Analysis

In order to study the microscopic characteristics and identify the presence of agglomerates larger than 1 µm, samples representing curcumin nanosuspension were observed with polarized light microscope (Leitz-Orthoplan, Leitz, Wetzlar, Germany). Observation was performed with magnification of 160 times.

#### 3.2.3. Zeta Potential (ZP) Determination

The surface charge of the curcumin nanosuspensions reflected by the zeta potential value were measured with Laser Doppler Anemometry using Zetasizer Nano ZS (Malvern Instruments Ltd., Malvern, UK). In order to study the adsorption characteristics of the stabilizers, freshly-prepared samples of all nanosuspensions were analyzed in two media: distilled water and original dispersion medium. Zeta potential was measured using electrophoresis mechanism, where the movement of charged particles was measured as electrophoresis mobility resulting from applied electrical field, referred as particle velocity [23]. The ZP was then calculated using the Smoluchowski equation:

$$\nu_{E}=\frac{\varepsilon_{0}\varepsilon_{r}\zeta }{µ}$$

In this equation, ν*_E_*, *ε*_0_, *ε_r_*, *ζ*, and µ respectively denote particle velocity, dielectric constant (kd) of solvent, vacuum permittivity, and dynamic viscosity of solution [23]. In this study, a field strength of 20 V/cm was used.

The potential of Stern layer, the immediate surface of nanocrystal, was determined by measuring the ZP of curcumin nanocrystal dispersed in double-distilled water which was supplemented with sodium chloride solution (0.9%) to achieve a conductivity of 50 µS/cm. Standard conductivity solution was used to minimize inconsistency in ZP value resulting from the solvent. The interference is commonly higher when the dispersed sample consists of electrolytes. The thickness of diffusion layer was measured by measuring ZP value in original dispersion medium. Higher ZP value was interpreted as indication of thicker diffusion layer and better stability of nanosuspension. The results can be used to predict long-term stability.

#### 3.2.4. Effect of Electrolytes on Physical Stability of Nanosuspensions

To study the effect of different stabilizers in the presence of electrolytes, CaCl_2_ 200 mM, simulated gastric fluid without enzyme (SGF, pH 1.2), and simulated intestinal fluid without enzyme (SIF, United States pharmacopoeia (USP) phosphate buffer solution, pH 6.8) were admixed to the nanosuspensions (NS), with a volume ratio of NS:electrolyte = 3:1. A solution of simulated gastric fluid was prepared with 0.7% (*v*/*v*) of hydrochloric acid adjusted to isotonicity using sodium chloride [24]. LD and polarized light microscopic analysis of the nanosuspensions were performed at 0, 30, 60, 120, and 240 min after electrolyte addition. In addition, ZP measurements and a simple visual settling rate observation were conducted to confirm the alteration of particle surface charge and particle size.

## Figures and Tables

**Table 1:** Zeta potential values of curcumin nanosuspension stabilized with five different stabilizers after addition of CaCl2 solution (150 mM), simulated gastric fluid (SGF), and simulated intestinal fluid (SIF).

#	Stabilizer	CaCl_2_ (mV)	SGF (mV)	SIF (mV)
1	PVA	−1.6	−1.2	−4.7
2	PVP	−7.4	−5.2	−20.3
3	TPGS	−8.9	−4.5	−19.6
4	SDS	−7.3	−19.1	−45.9
5	Na-CMC	−17.6	−9.8	−33.1

PVA: polyvinyl alcohol; PVP: polyvinyl pyrrolidone; TPGS: d-α-tocopheryl polyethylene glycol 1000 succinate; SDS: sodium dodecyl sulfate; Na-CMC: sodium carboxymethylcellulose.
